# Acetylated *Opuntia atropes* Mucilage: Physicochemical Characterisation and Application in Low-Fat Yogurt

**DOI:** 10.17113/ftb.64.03.26.9734

**Published:** 2026-09-15

**Authors:** Jaquelyn Alvarez-Marínez, Héctor Eduardo Martínez-Flores, Ma. Guadalupe Garnica-Romo, Rafael Zamora-Vega, Nelly Flores-Ramirez, Dalia Samanta Aguilar-Avila, Juan Pulido-Secundino, Salomon R. Vasquez-Garcia

**Affiliations:** 1 Facultad de Químico Farmacobiología. Programa Institucional de Maestría en Ciencias Biológicas, Universidad Michoacana de San Nicolás de Hidalgo, Tzintzuntzan 173. Col. Matamoros, 58240 Morelia, Mich., México; 2 Facultad de Químico Farmacobiología. Universidad Michoacana de San Nicolás de Hidalgo, Tzintzuntzan 173 Col. Matamoros, C.P., 58240 Morelia, Mich., México; 3 Facultad de Ingeniería Civil. Universidad Michoacana de San Nicolás de Hidalgo, Santiago Tapia 403. Col. Centro, 58000 Morelia, Mich., México; 4 Department of Wood Engineering and Technology, Universidad Michoacana de San Nicolás de Hidalgo, 58030 Morelia, Michoacán, México; 5 Universidad de Guadalajara, Centro Universitario de Ciencias Exactas e Ingenierías, Centro Universitario de Tlajomulco, Departamento de Ingeniería Química y Departamento de Clínicas, Guadalajara, Jalisco, México; 6 Centro Regional Universitario Centro Occidente (CRUCO), Universidad Autónoma de Chapingo, Periférico Paseo de la República No. 1000, Col. Lomas del Valle, 58170 Morelia, Mich. Mexico; 7 Department of Chemical Engineering, Universidad Michoacana de San Nicolás de Hidalgo, 58030 Morelia, Mexico

**Keywords:** cactus mucilage, hydrocolloids, polysaccharide functionalization, rheological properties, food stabilization, dairy products

## Abstract

**Research background:**

Mucilage extracted from *Opuntia atropes* cladodes is a plant-derived polysaccharide with promising techno-functional properties for food applications. Owing to its thickening and stabilizing capacity, cactus mucilage has attracted increasing interest as a natural ingredient in the formulation of reduced-fat dairy products. However, native mucilage may have functional limitations that could be improved through chemical modification. Nevertheless, limited information is available regarding the chemical modification of *O. atropes* mucilage and its application in fermented dairy systems.

**Experimental approach:**

In this study, mucilage was extracted from *O. atropes* cladodes and subsequently modified by acetylation to improve its functional properties. The extraction process was optimised to obtain mucilage efficiently while reducing solvent consumption. Native and acetylated mucilage samples were characterised through physicochemical analyses and structural evaluation using Fourier transform infrared spectroscopy (FTIR). Finally, the modified polysaccharide was incorporated into low-fat yogurt formulations to evaluate its effects on physicochemical, textural and stability properties.

**Results and conclusions:**

Acetylation modified the physicochemical and structural properties of the mucilage, affecting its hydration, viscosity and stability. Structural analysis confirmed the successful incorporation of acetyl groups into the polysaccharide matrix. When incorporated into low-fat yogurt formulations, the mucilage improved textural properties and reduced whey separation without adversely affecting key physicochemical parameters of the product. These results indicate that chemical modification can enhance the techno-functional performance of cactus mucilage in fermented dairy systems.

**Novelty and scientific contribution:**

This study offers new insights into the chemical modification of *O. atropes* mucilage and demonstrates its potential as a natural stabilizer in low-fat yogurt. The findings contribute to the development of plant-based hydrocolloids with enhanced functional properties and support the valorisation of cactus-derived polysaccharides as sustainable ingredients for the formulation of healthier dairy products.

## INTRODUCTION

Nopal (*Opuntia* spp.) is a member of the Cactaceae family, with more than 300 species worldwide. In Mexico, over 50 species are used as food and as raw material for medicinal products, while around 20 species are cultivated with varying degrees of domestication (*1*). Nopal is consumed for its fruit and for the whole or chopped cladode (nopalitos), which are the green, flattened, succulent stem segments of *Opuntia* plants, and is also used in animal feed and in industry to produce alcohol, dyes, soap, pectins and oils. Additionally, nopal has been reported to possess several medicinal properties, including hypoglycaemic effects associated with bioactive compounds such as polyphenols, dietary fibre and antioxidants, highlighting its potential relevance in the management of metabolic disorders and in health (*2*).

The chemical composition of nopal (*Opuntia* spp.) varies with factors such as species, environmental conditions and cultivation practices, with differences observed in nutrient and bioactive compound profiles across genotypes and tissues (*3*). It contains high levels of dietary fibre, protein, ash and lipids in species such as *Opuntia atropes*, *Opuntia hyptiacantha*, *Opuntia joconostle*, *Opuntia streptacantha* and *Opuntia tomentosa* (*4*). A significant fraction of the dietary fibre in Opuntia spp. comes from mucilage, a complex polysaccharide found in cladodes that is rich in galactose and arabinose and associated with the specialized cells of the cactus pads, reflecting its contribution to the plant’s structural and functional composition (*5*). Nopal mucilage has attracted interest in the food industry due to its functional properties; its composition, rich in sugars and uronic acids, allows it to form viscous gels and retain water and lipids, making it useful in foods targeted for people with diabetes or dyslipidaemia (*4*, *6*).

Mucilage from *Opuntia* cladodes consists primarily of high-molecular-mass heteropolysaccharides, consistently contains monosaccharides such as galactose, arabinose, xylose and rhamnose, with species-dependent quantity of galacturonic acid contributing to its complex structure (*7*). It exhibits a strong negative charge due to the dissociation of carboxyl groups. This chemical structure confers typical hydrocolloid behaviour, including water and oil absorption, formation of viscous gels, and retention of sugars and lipids, which are properties relevant to food applications.

However, despite its favorable techno-functional properties, native *Opuntia* mucilage may present limitations in solubility, structural stability and control of its rheological behaviour when incorporated into complex food matrices. The abundant hydroxyl groups present in polysaccharides provide opportunities for chemical modification, including etherification, acylation, acetylation, oxidation, silylation and hydrolysis (*8*). Among these approaches, acetylation involves the introduction of acetyl groups into hydroxyl functionalities, which can modify the hydrophilic–hydrophobic balance, intermolecular interactions, molecular conformation and functional properties of polysaccharides. The properties of the resulting derivatives depend on the nature of the acyl group introduced; thus, acetylation generally provides a moderate increase in hydrophobic character, whereas the incorporation of longer-chain acyl groups can produce more pronounced changes in hydrophobicity, solubility and interfacial characteristics. However, the potential effects of chemical modification on bioactive compounds associated with *Opuntia* mucilage, as well as on their functional properties, remain to be established. The technological relevance of acetylation has been demonstrated for other cactus-derived polysaccharides. For example, acetylated low-methoxyl pectins isolated from *Opuntia albicarpa* and *Opuntia matudae* exhibited shear-thinning behaviour and calcium-induced gel formation, while their acetyl groups did not impair gel development (*9*). More broadly, recent reviews indicate that chemical modification of cactus polysaccharides remains considerably less explored than their extraction and physicochemical characterisation, particularly regarding acetylation and its relationship with food functionality (*10*). However, these previous studies have focused mainly on pectins or other polysaccharide fractions from *Opuntia* fruits rather than on the controlled acetylation of cladode mucilage.

Previous studies have also demonstrated the potential of native *Opuntia* materials in dairy systems, although the available evidence has focused on applications different from the one investigated in the present study. Mucilage from *Opuntia ficus-indica* has been evaluated as a natural thickening agent in drinking yogurt, with different mucilage concentrations assessed in relation to pH, acidity, viscosity and sensory acceptance (*11*). Bernardino-Nicanor et al. (*12*) reported that supplementation of set-type yogurt with parenchymatous tissue powder from *Opuntia robusta*, which is rich in mucilage, increased water-related and functional properties and reduced the susceptibility of the yogurt to syneresis. In another approach, *O. ficus-indica* mucilage has been used as an encapsulating material for guava pulp intended for incorporation into yogurt; the resulting yogurt exhibited increased firmness, consistency and cohesion, together with enhanced dietary fibre and antioxidant-related properties (*13*). Furthermore, mucilage from *O. ficus-indica* and *O. atropes* has previously been added to raw milk, where reductions in mesophilic aerobic bacteria and total coliforms were observed (*14*). Collectively, these studies confirm the technological potential of *Opuntia*-derived materials in dairy matrices, but they also indicate that the reported applications have predominantly employed native mucilage, cladode tissue, or mucilage as an encapsulating material rather than chemically modified mucilage.

Yogurt was selected as the food matrix in the present study because it represents a particularly suitable system for evaluating the functionality of plant-derived hydrocolloids. Yogurt is a widely consumed fermented dairy product whose quality is strongly determined by the structure and stability of its protein-based gel network, with texture, viscosity, water-holding capacity and syneresis being important technological and sensory attributes (*15*, *16*). Hydrocolloids can interact with milk proteins and the aqueous phase, increase viscosity, improve water retention and modify gel structure, thereby reducing serum separation and improving textural characteristics (*16*, *17*). This functionality is especially relevant in low-fat yogurt because fat reduction can result in weaker body, lower firmness, poorer mouthfeel and increased susceptibility to syneresis, creating a technological need for alternative structuring and stabilizing ingredients (*16*, *18*). In addition, yogurt provides a consumer-accepted fermented food format in which natural, plant-derived ingredients can be incorporated without requiring the development of an entirely new food category. Recent research on plant-based stabilizers further supports the potential of natural mucilages and gums to improve the water-holding capacity, viscosity and textural properties of yogurt (*17*). Thus, yogurt, and particularly reduced-fat yogurt, provides a relevant model matrix in which the techno-functional effects of an acetylated *Opuntia* mucilage can be evaluated under realistic food conditions.

Taken together, the available literature indicates that the use of *Opuntia*-derived materials in yogurt has already been explored, but important knowledge gaps remain. No previous study has systematically investigated the controlled acetylation of mucilage isolated from *Opuntia atropes* cladodes, followed by a comprehensive evaluation of its physicochemical and structural properties and its application as a functional ingredient in low-fat yogurt. Moreover, the potential effects of acetylation on bioactive compounds associated with *Opuntia* mucilage and their functional properties remain to be established. The present work therefore differs from previous studies not only in the *Opuntia* species used, but also in the deliberate chemical modification of the mucilage before its incorporation into a dairy matrix. This approach enables the relationship between acetylation, mucilage functionality and yogurt performance to be investigated, thereby providing new information on the potential use of an underutilised *Opuntia* species as a source of modified food hydrocolloids.

Therefore, the objective of this study is to extract mucilage from *Opuntia atropes* cladodes, chemically modify it by acetylation at two levels using acetic anhydride, characterise its physicochemical and structural properties, and evaluate its application as a functional ingredient in low-fat yogurt. We hypothesize that controlled acetylation would modify selected techno-functional properties of the mucilage and improve the stability and textural characteristics of reduced-fat yogurt without adversely affecting its fundamental physicochemical parameters.

## MATERIALS AND METHODS

*Opuntia atropes* cladodes were collected at the Morelia Campus of the Autonomous University of Chapingo (CRUCO, Morelia, Michoacán, Mexico). The spines were removed from the cladodes, which were then washed with soap and water, cut into pieces of approx. 1 cm^2^, and blended with water at three ratios *V*(water):*m*(cladode)=8:1, 6:1 and 4:1. Thus, three separate extraction mixtures were prepared, corresponding to the three water:cladode ratios. Each mixture was heated to (83±2) °C for 2 h. For separation, the three samples were centrifuged using a Centrificient IV centrifuge (model TD5M-WS; CRM Globe Int., Mexico City, Mexico) at 1643×*g* for 15 min. The supernatants were transferred to an aluminium pot and concentrated by heating on a gas burner stove (model PFK-200; Koblenz-Lucero, Cuautitlán Izcalli, State of Mexico, Mexico) at 70 °C until their volume was reduced to one-third of the initial volume. Mucilage was then precipitated by adding 96 % ethanol (Meyer, Mexico City, Mexico) at ethanol volume ratios of 4:1 and 3:1), and the mixtures were allowed to stand at room temperature for 24 h. The precipitated mucilage obtained at each ethanol volume ratio was recovered separately by decantation and dried at 40 °C for 24 h in a digital oven (model 9053A; Ecoshel, Pharr, TX, USA). The mucilage extraction yield was calculated on a dry mass basis as the percentage ratio between the mass of dried mucilage recovered and the initial mass of fresh cladodes used for extraction, according to the following equation:


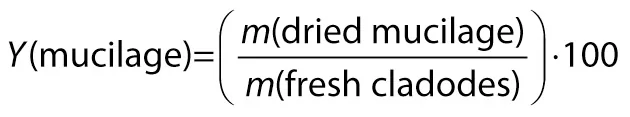
 /1/

Three extraction treatments were evaluated by combining the following water:cladode and ethanol:mucilage ratios 8:1+4:1, 6:1+3:1 and 4:1+3:1, respectively. After comparison of the extraction treatments, the mucilage obtained using the water:cladode and ethanol:mucilage ratios 4:1+3:1 treatment was selected for subsequent acetylation and yogurt preparation because it provided a comparable extraction yield while requiring lower amounts of water and ethanol.

### Chemical modification of Opuntia atropes mucilage by acetylation

*O. atropes* mucilage was acylated following the methods described by Huang *et al*. (*19*) and Prieto-Méndez *et al*. (*20*), with slight modifications. The mucilage obtained under the selected extraction conditions water:cladode and ethanol:mucilage ratios 4:1+3:1 was used as the native mucilage (NM) for subsequent acetylation. Two acetylation treatments were evaluated using 2 and 4 g of native mucilage while maintaining the same volume of acetic anhydride (23.2 mL) in both treatments. Thus, three mucilage samples were obtained for subsequent characterisation: NM, acetylated mucilage (AM) 1.78 % and AM 2.28 %. Both acetylated mucilages were characterised to evaluate the effect of the acetylation level on their physicochemical and functional properties. For yogurt formulation, NM and AM 1.78 % were selected to evaluate the effect of a moderately acetylated mucilage on yogurt properties while avoiding the use of the higher acetylation level (AM 2.28 %).

In the first treatment, 2 g of mucilage were reacted with 23.2 mL of acetic anhydride, giving an acetic anhydride:mucilage ratio of 11.6 mL/g and yielding acetylated mucilage (AM) with an acetylation level of 2.28 % (AM 2.28 %). In the second treatment, 4 g of mucilage were reacted with the same volume (23.2 mL) of acetic anhydride, giving a ratio of 5.8 mL/g and yielding acetylated mucilage with an acetylation level of 1.78 % (AM 1.78 %).

In both treatments, the mucilage was first dispersed in 75 % ethanol and stirred for 10 min at room temperature. The pH of the dispersion was then adjusted to 8.0 before acetic anhydride (Meyer) was added, and the reaction was allowed to proceed for 10 min. Subsequently, the pH was adjusted to 4.5. The acetylated mucilage was recovered by centrifugation (Centrificient IV model TD5M-WS; CRM Globe Int.) at 1643×g for 15 min and washed three times with 75 % ethanol. Finally, the recovered mucilage was dried at 40 °C for 24 h in a digital oven (model 9053A; Ecoshel), ground, and sieved through a US #120 mesh (USA Standard Testing Sieve ASTM Specification E-11, Daigger Scientific, Vernon Hills, IL, USA).

The yield of acetylated mucilage was determined by weighing the dried product recovered after the acetylation and drying steps. The recovered mass was reported as the yield (g). The yield was calculated as the percentage ratio between the mass of dried acetylated mucilage recovered and the initial mass of native mucilage used in each reaction (2 or 4 g), multiplied by 100, according to the following equation:


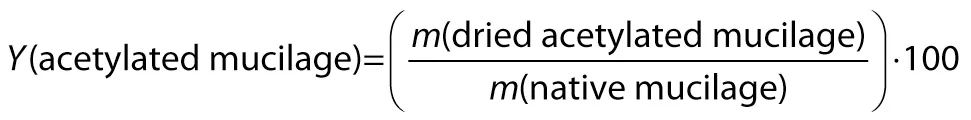
 /2/

Thus, three mucilage samples were obtained for subsequent characterisation: NM, AM 1.78 % and AM 2.28 %. For yogurt preparation, NM and AM 1.78 % were selected for subsequent formulation.

### Determination of acetylation percentage and degree of substitution

The acetyl content and degree of substitution (DS) of the acetylated mucilage were determined according to the method described by Vargas *et al*. (*21*), with some modifications. Briefly, 0.5 g of acetylated mucilage was placed in a 250-mL Erlenmeyer flask, followed by the addition of 25 mL of distilled water and three drops of phenolphthalein indicator (Hycel Mexico, S.A. de C.V., Zapopan, Mexico). Subsequently, 12.5 mL of 0.45 M NaOH solution (Jalmek Cientifica, San Nicolás de los Garza, N.L., Mexico) were added. The mixtures were shaken for 30 min and titrated with 0.2 M HCl solution (Meyer). A blank was prepared simultaneously using native mucilage and was titrated under the same conditions.

The acetyl content (w/%) is the percentage by mass of acetyl groups in the dry mucilage and was calculated using the following equation:


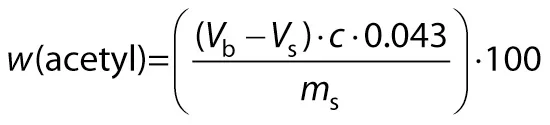
 /3/

where *V*_b_ is the volume of HCl used for the blank titration (mL), *V*_s_ is the volume of HCl used for the sample titration (mL), *c* is the concentration of the HCl solution (0.2 M), 0.043 is the mass of acetyl groups corresponding to 1 mL of 1 M HCl (g/mL) calculated from the molar mass of the acetyl group (43.045 g/mol) and *m*_s_ is the dry mass of the mucilage sample (g).

The degree of substitution (DS) was calculated using the following equation:


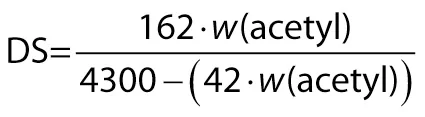
 /4/

where 162 is the molecular mass of an anhydroglucose unit (g/mol), 4300 is the molecular mass of the acetyl group multiplied by 100, and 42 is the molecular mass of the acetyl group minus one hydrogen atom (g/mol).

### Physicochemical analyses of mucilage

The following physicochemical properties of native and acetylated mucilage were evaluated: pH, colour parameters, water activity (*a*_w_), water and oil absorption capacities (WAC and OAC) and viscosity.

For pH determination, solutions were prepared by dispersing 0.5 g of native or acetylated mucilage in 5 mL of distilled water. The pH was measured using a digital pH metre (Aquasearcher A-AB23PH; OHAUS, Parsippany–Troy Hills, NJ, USA). Colour of the mucilage powders was measured using a BYK Gardner colourimeter (model CC-6801; BYK Gardner, Columbia, MD, USA) in the CIELAB colour space. The evaluated parameters included L* (lightness), a* (red-green axis), b* (yellow-blue axis), chroma (C*) and hue angle (*h*°). The L* parameter represents lightness (0=black, 100=white), a* indicates red (positive) or green (negative) tones, and b* represents yellow (positive) or blue (negative) tones. Chroma (C*) describes colour saturation, while hue angle (*h*°) corresponds to the position of the colour within the chromatic circle (0°=red, 90°=yellow, 180°=green, 270°=blue). Water activity (*a*_w_) was measured in triplicate using a water activity metre (model LabSwift-aw; Novasina AG, Lachen, Switzerland). Water absorption capacity (WAC) was determined following the methodology described by Bocarando-Guzman *et al*. (*22*). Briefly, 0.3 g of mucilage powder was placed in 50-mL conical tubes (BluCappTM, Nordhausen, Germany) with 4.5 mL of distilled water, vortexed for 10 min, and then shaken on an orbital shaker (model CVP-2000P; CScientific, Mexico City, Mexico) for 1 h. The samples were centrifuged at 2146×*g* for 10 min, the supernatant was decanted, and the hydrated mucilage was weighed. WAC (g water per g mucilage) was calculated using the following equation:


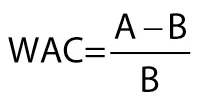
 /5/

where A represents the mass of mucilage with absorbed water (g), and B corresponds to the mass of dry mucilage (g). Oil absorption capacity (OAC) was determined using the same procedure, replacing distilled water with canola oil.

Viscosity was measured following the method described by González-Sandoval *et al.* (*23*), with slight modifications. Native and acetylated mucilage powders were dispersed in distilled water at a ratio of 1:100 and stirred at 600 rpm for 24 h at room temperature using a hot plate with a stirrer (model Guardian 7000; OHAUS). A volume of 10 mL of each solution was then transferred to a viscometre (model DV3T; Brookfield/AMETEK, Middleboro, MA, USA) equipped with a ULA (0) spindle. Measurements were taken at increasing rotational speeds (50, 75 and 100 rpm), ensuring a torque of ≥10 %. All measurements were conducted at room temperature in triplicate.

### Analysis of the chemical composition of mucilage

Protein, ash and calcium contents of native mucilage (NM) and the two acetylated mucilage samples obtained from the acetylation treatments using acetic anhydride ratios of 5.8 and 11.6 mL/g were determined in triplicate. These treatments yielded acetylation percentages of 1.78 % (AM 1.78 %) and 2.28 % (AM 2.28 %), respectively. Protein was measured using AOAC 955.04/90 Kjeldahl method (*24*) with a conversion factor of 6.25, and ash content was determined using AOAC 942.05/90 method (*24*). For calcium analysis, ash obtained by incineration was wet-digested, solubilized and, if necessary, diluted to fall within the linear range of the instrument. Calcium was quantified by flame atomic absorption spectrometre (AAnalyst 200; PerkinElmer, Inc., Waltham, MA, USA) using a calibration curve with certified standards (20–80 mg/g). Accuracy was confirmed with a reference standard of 80 mg/g, which yielded a measured value of 80.5 mg/g.

### Fourier transform infrared spectroscopy analysis of mucilage

Fourier transform infrared-attenuated total reflectance (FTIR-ATR) spectroscopy was used to identify functional groups in native and acetylated (1.78 and 2.28 %) *Opuntia atropes* mucilages. Analyses were conducted using FTIR equipment (model Vertex 70/70 V; Bruker, Ettlingen, Germany) equipped with an ATR accessory. Spectra were recorded over the range 400–4000 cm^−1^, with a resolution of 4 cm^−1^ and a scan time of 32 s.

### Yogurt preparation

Among the two acetylated mucilage samples, AM 1.78 % (mucilage with an acetyl content of 1.78 %) was selected for yogurt formulation based on its acetylation degree and physicochemical and functional properties, while AM 2.28 % was retained for characterisation to evaluate the effect of increasing acetylation on mucilage properties. Gelatine (Coloidales Duché, S.A. de C.V., Mexico City, Mexico) was incorporated into selected yogurt formulations as a conventional gelling/thickening ingredient to assess whether its addition produced an additional or synergistic effect on yogurt structural properties, particularly viscosity and syneresis, when combined with native or acetylated mucilage. Five yogurt formulations were prepared: control yogurt (control), yogurt containing native mucilage (NM), yogurt containing native mucilage and gelatine (NM+Gel), yogurt containing mucilage acetylated to 1.78 % (Acet M), and yogurt containing mucilage acetylated to 1.78 % and gelatine (Acet M+Gel). Yogurts were prepared as follows: the starter culture Lyo Pro Y Mild (Codex-Ing Biotech Ingredients, Miami, FL, USA), composed of *Streptococcus salivarius* ssp. *thermophilus* and *Lactobacillus delbrueckii* ssp. *bulgaricus*, was reactivated from a freeze-dried preparation by reconstitution in low-fat milk (Santa Clara Light®, Pachuca, Hgo., Mexico) to obtain a stock suspension for inoculation. For the control yogurt, 100 mL of low-fat milk were inoculated with 1 mL of stock suspension. For NM and Acet M yogurts, 0.5 g of native mucilage or mucilage acetylated to 1.78 %, respectively, was added to 100 mL of low-fat milk. For NM+Gel and Acet M+Gel yogurts, 0.5 g of native mucilage or mucilage acetylated to 1.78 %, respectively, together with 0.5 g of gelatine was added to 100 mL of low-fat milk. All formulations were then inoculated with 1 mL of the stock suspension. All mixtures were incubated at 43 °C for 8 h in an incubator (model LSIK-B2V/1C111; Incucell MMM Group, MMM Medcenter Einrichtungen GmbH, Munich, Germany) to allow fermentation and obtain the final yogurt products. The yogurt formulations prepared and analysed are summarised in Table 1.

**Table 1:** Formulation of the yogurt samples

Yogurt sample	*m*(mucilage)/g	*m*(acetylated mucilage)/g	*m*(gelatine)/g
Control yogurt	-	-	-
Native mucilage yogurt	0.5	-	-
Native mucilage+gelatine yogurt	0.5	-	0.5
Acetylated mucilage yogurt	-	0.5	-
Acetylated mucilage+gelatine yogurt	-	0.5	0.5

AM=mucilage acetylated to a degree of 1.78 %

The physicochemical properties of the yogurt treatments-control, yogurt with native mucilage, yogurt with native mucilage and gelatine, yogurt with 1.78 % acetylated mucilage, and yogurt with 1.78 % acetylated mucilage and gelatine, were evaluated as follows: 10 mL samples from each treatment were placed in beakers at 8 °C and pH was measured using a digital benchtop pH metre (Aquasearcher A-AB23PH; OHAUS, Parsippany-Troy Hills, NJ, USA). Colour parameters (L*, a*, b*, C* and *h*°) were determined in triplicate using a BYK Gardner colour and brightness colourimeter (BYK Gardner, Columbia, MD, USA). Water activity was measured in triplicate using a water activity metre (LabSwift-aw; Novasina AG, Lachen, Switzerland). Viscosity of 10 mL samples from each treatment maintained at 8 °C prior to analysis was determined using a Brookfield DV3T viscometer (Brookfield/AMETEK) with the ultra-low adapter (ULA) (0) spindle at 10 rpm, following the method described in González-Sandoval *et al*. (*23*) with minor modifications. The degree of syneresis was determined in triplicate using 20 g of each sample centrifuged (Centrificient IV model TD5M-WS; CRM Globe Int.) at 1643×*g* for 15 min at room temperature. Syneresis (%) was calculated as follows:


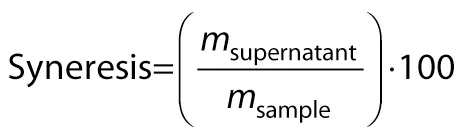
 /6/

where *m*_supernatant_ is the mass of the supernatant recovered after centrifugation (g) and *m*_sample_ is the initial mass of the yogurt sample before centrifugation (20 g).

Titratable acidity was determined in triplicate as lactic acid according to NOM-185-SSA1-2002 (*25*). Lactic acid content (%) was calculated using the following formula:


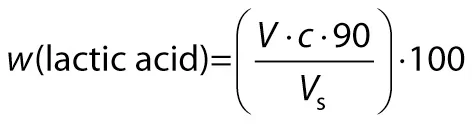
 /7/

where *V* is the volume of NaOH solution used (L), *c* is the concentration of NaOH (0.1 M), *V*_s_ is the volume of sample (mL), and 90 is the molar mass of lactic acid (g/mol).

### Statistical analysis

All experiments were conducted in triplicate (*N*=3), and the results are expressed as mean±standard deviation. Statistical analyses were carried out using R v. 4.3.2 (*26*) through the RStudio IDE v. 2023.12.0 (Build 369; Posit Software, PBC) (*27*). For experiments involving three or more treatment groups, a one-way analysis of variance (ANOVA) was used to evaluate differences among treatment means. When the ANOVA indicated a significant treatment effect (p<0.05), Tukey’s honestly significant difference (HSD) test was used for multiple pairwise comparisons. For comparisons involving only two treatment groups, statistical significance was assessed using the F-test from the one-way ANOVA. With two groups, the one-way ANOVA F-test is mathematically equivalent to Student’s *t*-test for two independent groups (F=t^2^); therefore, no *post-hoc* multiple-comparison test was required. Statistical significance was defined as p<0.05.

## RESULTS AND DISCUSSION

### Mucilage extraction from Opuntia atropes

The results of mucilage extraction from 1 kg of *Opuntia atropes* cladodes are shown in Table 2. No statistically significant differences were observed among the extraction treatments. Nevertheless, the water:cladode and ethanol:mucilage 4:1+3:1 treatment was selected for subsequent experiments because it provided a comparable extraction yield while requiring smaller volumes of water and ethanol. Thus, this treatment represented the most resource-efficient condition among those evaluated. Extraction yield is influenced by the volumes of water and ethanol used, as well as by extraction temperature, cladode species and age, and cultivation conditions (*28*).

**Table 2:** Yield of mucilage extraction from *Opuntia atropes*

Water:cladode ratio	Ethanol:mucilage ratio	*Y*/%
8:1	4:1	(1.0±0.2)^a^
6:1	3:1	(0.8±0.2)^a^
4:1	3:1	(0.9±0.1)^a^

Different lowercase letters in superscript indicate significant differences among treatments according to one-way ANOVA followed by Tukey’s HSD test (p<0.05). Values are expressed as mean±standard deviation (*N*=3)

### Acetylation percentage and degree of substitution of acetylated mucilages of Opuntia atropes

The acetylation of native mucilage was evaluated under two experimental conditions using acetic anhydride:mucilage ratios of 11.6 and 5.8 mL/g. Table 3 summarises the reaction yields, acetyl group content and degree of substitution (DS). The treatment using 2 g of mucilage showed a higher acetylation percentage (2.28 %) and DS (0.088), while the 4 g treatment resulted in 1.78 % acetylation and a DS of 0.068. These differences can be attributed to the reagent–substrate ratio (*19*, *20*). In both treatments, the same volume of acetic anhydride was used, resulting in ratios of 11.6 mL/g for the 2 g treatment and 5.8 mL/g for the 4 g treatment. Increasing the mucilage mass without changing the reagent volume reduced the amount of acetic anhydride available per gram of substrate, which explains the lower acetylation degree and DS observed in the 4 g treatment. A higher liquid-to-solid ratio enhances reagent availability and may lead to higher substitution degrees, a phenomenon previously reported for polysaccharide modification processes (*29*, *30*).

**Table 3:** Yields, acetylation percentage and degree of substitution of acetylated mucilages of *Opuntia atropes*

(*V*(acetic anhydride):*m*(mucilage))/(mL/g)	*m*(recovered acetylated mucilage)/g	*Y*/%	Acetylation/%	DS
11.6	(1.4±0.2)^b^	(70.0±8.5)^b^	(2.28±0.07)^a^	(0.09±0.01)^a^
5.8	(3.30±0.02)^a^	(82.5±0.7)^a^	(1.8±0.1)^b^	(0.07±0.01)^b^

Different lowercase letters in superscript within the same column indicate significant differences between treatments according to the F-test from one-way ANOVA (p<0.05). For two treatment groups, the F-test is mathematically equivalent to Student’s *t*-test. Values are expressed as mean±standard deviation (*N*=3). DS=degree of substitution

From a functional perspective, incorporating acetyl groups into the polysaccharide structure partially replaces hydroxyl groups, reduces intermolecular hydrogen bonding and alters the hydrophilic–hydrophobic balance of the polymer. As a result, acetylation can modify properties such as solubility, viscosity, water retention capacity and interactions with other food components. In general, moderate DS values, such as those obtained in this study, may improve the functional performance of polysaccharides as stabilizers or thickening agents in food systems, while preserving their compatibility with aqueous matrices.

However, increasing the reagent concentration may also risk exceeding regulatory limits commonly set for chemically modified food polysaccharides. For example, modified starches are generally limited to 2.5 % acetyl groups and a DS of 0.1 (*31*-*33*). Although specific regulations for plant mucilages such as nopal mucilage are not currently established in Mexican or Codex standards, these references can serve as useful comparative guidelines.

### Physicochemical properties and chemical composition of native and acetylated mucilage

Table 4 summarises the physicochemical properties of native and acetylated mucilage samples. Native mucilage exhibited a near-neutral pH (6.60), whereas acetylated mucilage showed significantly lower values (4.97±0.01). This decrease is likely associated with the incorporation of acetyl groups, the presence of residual acetic acid, and the pH adjustment performed during the modification process. A slightly acidic pH may influence the functional behaviour of mucilage in food systems (*6*).

**Table 4:** Physicochemical properties of native and acetylated mucilages of *Opuntia atropes* obtained using the selected extraction treatment (4:1 water:cladode; 3:1 ethanol:mucilage treatment)

		Mucilage acetylation degree/%	
Parameter	Native mucilage	1.78	2.28
pH	(6.60±0.01)^a^	(4.97±0.01)^b^	(4.97±0.01)^b^
L*	(63.1±1.3)^a^	(50.1±2.3)^b^	(48.4±1.9)^c^
a*	(−1.50±0.02)^a^	(−1.45±0.06)^b^	(−1.2±0.2)^c^
b*	(9.0±0.2)^c^	(13.8±0.8)^a^	(11.4±0.5)^b^
C*	(9.3±0.3)^c^	(14.1±0.6)^a^	(11.9±1.4)^b^
*h*°	(98.3±0.8)	(95.7±0.3)^b^	(95.2±0.8)^c^
*a*_w_	(0.41±0.01)^a^	(0.28±0.04)^c^	(0.31±0.01)^b^
WAC/(*m*(water)/ *m*(mucilage)/(g/g)	(7.1±0.2)^b^	(10.3±0.2)^a^	(10.3±0.3)^a^
OAC/(*m*(oil)/*m*(mucilage)/(g/g)	(2.5±0.3)^ns^	(2.2±0.1)^ns^	(2.05±0.07)^ns^
*η*/(Pa·s)	(0.0131±0.0004)^b^	(0.010±0.001)^c^	(0.0178±0.0006)ª

Different lowercase letters in superscript within the same row indicate significant differences among treatments according to one-way ANOVA followed by Tukey’s HSD test (p<0.05). ns=no significant difference among treatments. Values are expressed as mean±standard deviation (*N*=3). WAC=water absorption capacity, OAC=oil absorption capacity

Changes in colour parameters were also observed after acetylation. The L* value decreased, indicating a darker appearance, with a tendency to decline as the degree of acetylation increased. All samples exhibited slightly greenish (a*) and yellowish (b*) tones that became more pronounced after modification. In addition, acetylated mucilages showed higher chroma (C*), indicating greater colour saturation, while the hue angle (*h*°) approached 90°, corresponding to yellowish tones. These variations may be related to structural modifications in the polysaccharide network caused by the introduction of acetyl groups, which can alter light scattering properties (*34*).

Acetylation also influenced the hydration behaviour of the mucilage. Native mucilage exhibited an *a*_w_ of 0.41±0.01, whereas the acetylated samples showed significantly lower values (0.28±0.04 for 1.78 % acetylation and 0.31±0.01 for 2.28 %). The reduction in *a*_w_ suggests modifications in the water-binding capacity of the polymer matrix, likely related to the partial substitution of hydroxyl groups with acetyl groups, which decreases the number of sites available for hydrogen bonding with water molecules (*29*). These low *a*_w_ values indicate high stability and low susceptibility to microbial growth, consistent with previous observations for cactus mucilage (*35*).

Despite the reduction in water activity, acetylation significantly increased the water absorption capacity (WAC). Native mucilage exhibited a WAC of 7.1±0.2, while acetylated samples reached 10.3±0.2 (1.78 %) and 10.3±0.3 (2.28 %). This behaviour suggests structural rearrangements in the polymer network that favour water uptake, possibly through the formation of a more open matrix and the weakening of intermolecular interactions. Similar trends have been reported for modified polysaccharides, where structural expansion facilitates water penetration into the polymer network (*36*, *37*).

Oil absorption capacity (OAC), in contrast, did not differ significantly among treatments, with values ranging from 2.05 to 2.51 g oil per g mucilage. This indicates that the degree of substitution achieved was not sufficient to substantially modify hydrophobic interactions within the polymer matrix. Nevertheless, the moderate OAC values observed suggest potential applications in food formulations requiring emulsifying or stabilizing agents.

The structural modifications induced by acetylation were also reflected in viscosity behaviour. Native mucilage showed a viscosity of (0.0131±0.0004) Pa·s. Acetylation at 1.78 % reduced viscosity to (0.010±0.001) Pa·s, likely due to decreased polarity and weaker intermolecular interactions following the introduction of acetyl groups. However, the sample acetylated at 2.28 % exhibited a marked increase in viscosity to (0.0178±0.0006) Pa·s, suggesting structural rearrangement and the formation of a more compact polymer network at higher substitution levels. Similar non-linear effects of acetylation on viscosity have been reported for modified polysaccharides (*38*).

The compositional analysis (Table 5) provides additional insight into the observed physicochemical behaviour. Protein mass fraction differed significantly among samples, increasing from (9.2±0.3) % in native mucilage to (11.5±0.2) % in the sample with a DS≈0.068, then decreasing to (7.5±0.2) % at DS≈0.088. The initial increase may reflect greater retention of proteins associated with the polysaccharide matrix due to structural changes induced by acetylation, whereas the subsequent decrease could be related to the removal of loosely bound proteins during the washing steps of the modification process. Reports on protein content in acetylated nopal mucilage are scarce; therefore, these findings provide novel information on the compositional changes associated with this chemical modification.

**Table 5:** Protein, ash and calcium mass fractions of native and acetylated *Opuntia atropes* mucilages obtained using the selected extraction treatment (water:cladode and ethanol:mucilage 4:1+3:1)

		Mucilage acetylation degree/%	
Chemical component	Native mucilage	1.78	2.28
*w*(protein)/%	(9.2±0.3)^b^	(11.5±0.2)^a^	(7.5± 0.2)^c^
*w*(ash)/%	(9.4±0.2)^b^	(13.0±0.1)^a^	(11.5±1.3)^a^
*w*(calcium)/(mg/g)	(87.5±1.0)^c^	(161.8±3.5)^a^	(92.0±2.0)^b^

Different superscript lowercase letters within the same row indicate significant differences among treatments according to one-way ANOVA followed by Tukey’s HSD test (p<0.05). ns=no significant difference among treatments. Values are expressed as mean±standard deviation (*N*=3)

Ash mass fraction also increased after acetylation, from (9.4±0.2) % in native mucilage to (13.0±0.1) and (11.5±1.3) % in the acetylated samples. Chemical modification of starch via acetylation can significantly alter its chemical composition, including mineral content, as part of its overall physicochemical transformation (*39*), and may result from the retention of mineral compounds or residual salts generated in the alkaline reaction medium.

Mineral analysis revealed notable differences in calcium mass fractions. The mucilage acetylated at 1.78 % (DS≈0.068) had the highest calcium mass fraction ((161.8±3.5) mg/g), followed by the sample acetylated at 2.28 % ((92.0±2.0) mg/g) and the native mucilage ((87.5±1.0) mg/g). These results suggest that acetylation alters the interaction between the polysaccharide matrix and divalent cations. Mucilage from *Opuntia* species contains uronic acids that provide carboxyl groups capable of binding Ca^2+^ through ionic interactions within the polymer network (*40*, *41*). Moderate structural modification may favour the exposure or reorganization of these binding sites, enhancing calcium retention, whereas higher substitution degrees may reduce the availability of hydrophilic groups involved in cation binding.

The treatment with the highest calcium mass fraction also showed higher ash mass fraction and greater water absorption capacity, suggesting that calcium ions may contribute to water retention through ionic interactions with the polysaccharide matrix. Such interactions can promote the formation of junction zones within the polymer network, affecting hydration and structural organization without necessarily increasing viscosity (*42*).

Overall, the combined physicochemical and compositional results indicate that acetylation modifies the structural organization of the mucilage polysaccharide network, affecting polymer–water interactions, mineral binding and rheological behaviour. These structural changes influence hydration capacity, viscosity and stability, which are key functional attributes for hydrocolloid applications. Consequently, both native and acetylated mucilage from O. atropes show potential as natural hydrocolloids with applications as stabilizers, thickeners or texture modifiers in complex food systems, including fermented dairy products such as yogurt.

### Fourier transform infrared spectroscopy analysis

Fourier transform infrared (FTIR) analysis enabled the identification of the main functional groups present in native mucilage (NM) and in mucilages chemically modified by acetylation at 1.78 % (AM 1.78 %) and 2.28 % (AM 2.28 %). Fig. 1 shows the FTIR spectra of the samples, where characteristic bands of polysaccharide compounds were detected, with variations attributed to structural modifications induced by acetylation.

**Fig. 1:**
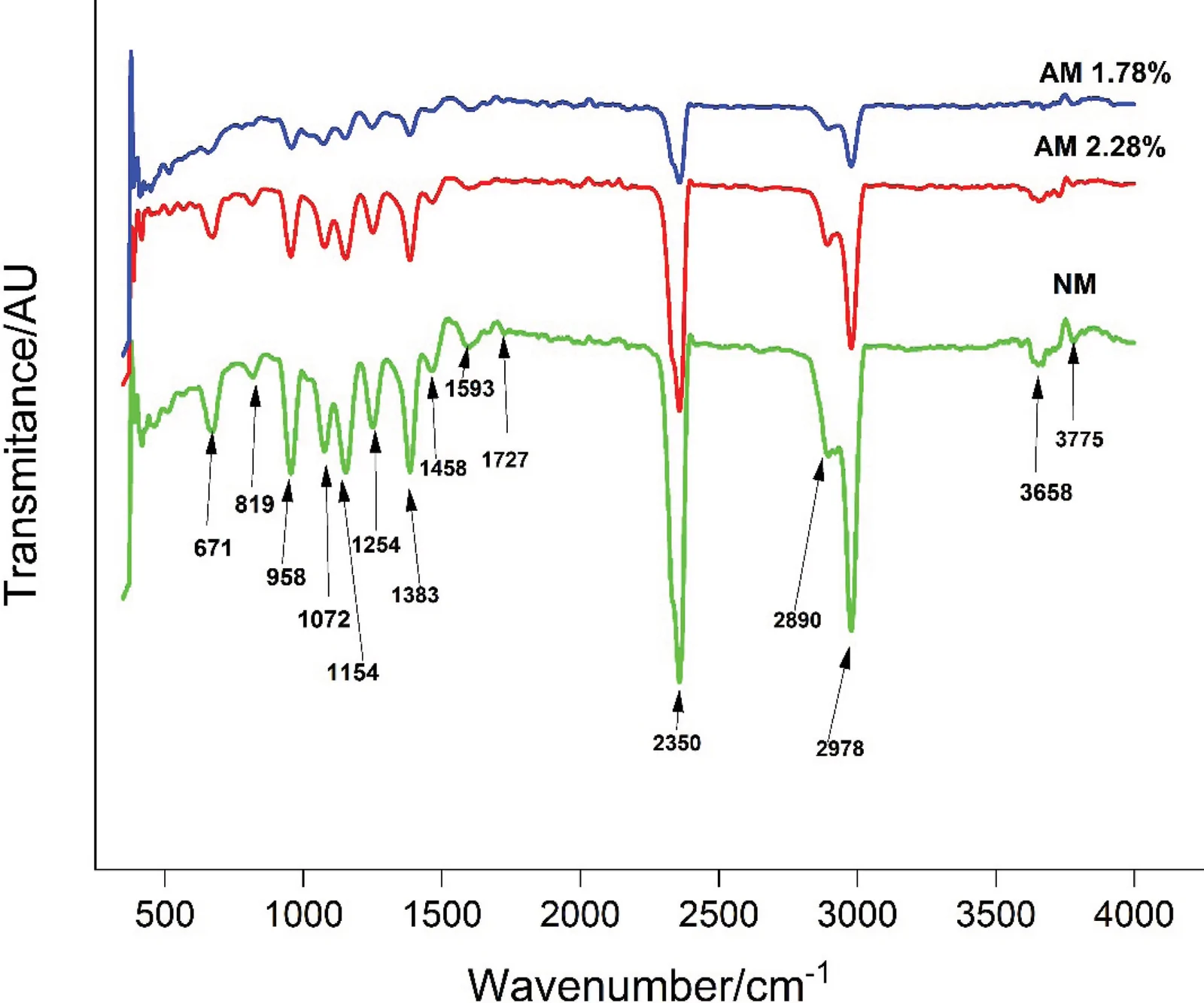
Spectra of native mucilage (NM), mucilage acetylated to 1.78 % (AM 1.78 %) and mucilage acetylated to 2.28 % (AM 2.28 %) of *Opuntia atropes* obtained by Fourier transform infrared spectroscopy (FTIR)

A band at 2978 cm−1 was observed, corresponding to the symmetric and asymmetric stretching vibrations of C–H groups associated with methylene and methyl groups in sugar structures. This result is consistent with that reported by Rodríguez-Gonzalez *et al*. (*6*), who observed the same band with a shift at 2933 cm^-1^. These vibrations are present in the hydrocarbon backbone of the polysaccharide chains as well as in the acetyl groups. The higher intensity of these bands in AM 1.78 % and NM than in AM 2.28 % may be attributed to improved molecular orientation and stronger vibrational coupling rather than to an increase in C–H content (*43*).

In *Opuntia* mucilage, a band at 1630 cm^−1^ is commonly observed and is associated with uronic acids (COO^−^ groups) derived from pectins (*40*). In the present spectra, a weak band at 1727 cm^−1^ corresponding to C=O stretching vibrations was detected, which may be attributed to acetyl groups forming ester linkages within the polysaccharide structure. Additionally, a band was observed at 1593 cm^−1^. In the spectra of the acetylated mucilage, faint undulations were detected in this region; however, their low intensity may be related to secondary structural effects, particularly in AM 2.28 %, where higher intensity might be expected due to the greater degree of substitution. Similar observations were reported by Sun *et al*. (*44*), who indicated that the band near 1727 cm^−1^ may weaken or even disappear depending on the chemical environment of the polymer, especially in hydrophilic media or when the content of ester groups is low, which directly affects the polarity and absorbance of the C=O bond. Despite the low intensity of this band, chemical modification was quantitatively confirmed by the titration of acetyl groups and the degree of substitution values presented in Table 3, supporting the presence of acetyl groups in the polymer structure.

In the 1450–1370 cm^−1^ region, bending vibrations associated with –CH3 (methyl) and –CH2– (methylene) groups were identified. These bands are related both to the structural units of the polysaccharides and to the incorporation of acetyl groups (–COCH3) into the polysaccharide network. A slight increase in intensity was observed for AM 1.78 %, which may reflect a balance between chemical substitution and structural preservation. In contrast, the signal was weaker for AM 2.28 %, possibly due to increased structural disorder or reduced crystalline order, effects previously reported in chemically modified cellulose systems (*45*, *46*).

Signals around 1254 cm^−1^ were also detected and are attributed to δC–H, δCH2 and δO–H deformation vibrations typical of mucilaginous polysaccharides, providing additional information on the vibrational behaviour of the polymeric structure (*47*). In this region, a secondary contribution associated with acetyl groups linked to the polysaccharide backbone may also be present.

The region between 1200 and 800 cm^−1^, commonly referred to as the polysaccharide fingerprint region, exhibited several characteristic vibrations associated with stretching of carbohydrate structures (*48*). These include bands attributed to C–O–C glycosidic linkages at 1154 cm^−1^, C–O stretching of alcohol groups at 1072 cm^−1^ and vibrations related to sugar rings and β-glycosidic bonds around 819 cm^−1^. For AM 2.28 %, this region showed a noticeable decrease in band definition, suggesting greater structural modification and loss of molecular order. In contrast, NM and AM 1.78 % displayed more clearly defined bands, indicating the preservation of a partially ordered structure.

Overall, the FTIR results confirm the partial incorporation of acetyl groups into the mucilage structure. Comparison of the spectra indicates that acetylation at 1.78 % largely preserved the structural organization of the polysaccharide, whereas acetylation at 2.28 % induced more pronounced changes in molecular arrangement, reflected in the reduced definition of key spectral regions. These findings support the effectiveness of the chemical modification process and its impact on the structural properties of the mucilage.

### Physicochemical properties of yogurt

The physicochemical properties evaluated for the yogurt formulations were pH, colour parameters, water activity (*a*_w_), viscosity, degree of syneresis and titratable acidity (Table 6) to determine the effect of incorporating native mucilage or mucilage with 1.78 % acetylation, added at 0.5 g per 100 mL of milk, alone or in combination with gelatine.

**Table 6:** Physicochemical properties of yogurts

	Yogurt				
Parameter	Control	NM	NM+Gel	Acet M	Acet M+Gel
pH	(4.57±0.01)^a^	(4.47±0.01)^b^	(4.47±0.01)^b^	(4.47±0.00)^b^	(4.47±0.01)^b^
L*	(67.9±3.0)^bc^	(66.4±2.1)^c^	(64.1±2.5)^c^	(73.6±0.3)^a^	(67.2±0.5)^b^
a*	(-1.4±0.2)^a^	(-2.00±0.07)^c^	(-2.0±0.4)^c^	(-1.82±0.01)^b^	(-1.56±0.02)^a^
b*	(2.5±0.3)^c^	(4.8±0.2)^a^	(5.0±0.1)^a^	(3.60±0.05)^b^	(3.32±0.01)^b^
C*	(3.5±0.4)^c^	(5.2±0.2)^a^	(5.44±0.06)^a^	(4.08±0.01)^b^	(3.68±0.02)^c^
*h*°	(117.7±4.5)^a^	(112.8±0.5)^c^	(110.2±0.9)^c^	(116.4±0.3)^b^	(115.3±0.2)^b^
*a*_w_	(0.98±0.00)^ns^	(0.98±0.00)^ns^	(0.98±0.00)^ns^	(0.98±0.00)^ns^	(0.98±0.00)^ns^
*η*/cP	(198.2±8.7)^a^	(236.6±1.9)^c^	(392.7±8.7)^e^	(218.4±7.6)^b^	(308.3±1.0)^d^
Syneresis/%	(84.4±1.0)^a^	(82.7±0.6)^a^	(82.3±0.9)^ab^	(80.4±0.3)^c^	(79.9±0.4)^c^
TA/%	(0.83±0.01)^a^	(0.86±0.01)^b^	(0.84±0.01)^ab^	(0.82±0.01)^a^	(0.87±0.01)^c^

Different lowercase letters in superscript within the same row indicate significant differences among treatments according to one-way ANOVA followed by Tukey’s HSD test (p<0.05). ns=no significant difference among treatments. Values are expressed as mean±standard deviation (*N*=3). NM=native mucilage, NM+Gel=native mucilage and gelatine, Acet M=mucilage acetylated to 1.78 %, Acet M+Gel=mucilage acetylated to 1.78 % and gelatine, TA=titratable acidity

The control yogurt exhibited a pH=4.57±0.01, which was significantly higher (p<0.05) than that of the other formulations. Yogurts containing native mucilage and mucilage with 1.78 % acetylation (0.5 g/100 mL) showed similar pH values (4.47±0.01 and 4.47±0.00, respectively), with no significant differences between them, suggesting that the acetylation process did not significantly affect the acidity of the formulations. This behaviour may be attributed to the slightly acidic nature of O. atropes mucilage and its limited buffering capacity, which is not sufficient to significantly modify the final pH of the yogurt matrix (*28*, *49*). The formulation containing native mucilage combined with gelatine showed a slightly higher pH (4.49), possibly due to the amphoteric nature of gelatine and its interaction with acidic and basic components in the system, owing to the presence of both amino and carboxyl groups along the gelatine peptide chains that can carry variable charge depending on environmental pH (*50*). Despite these differences, all samples remained within the optimal pH range for yogurt (4.0–4.6).

Colour analysis revealed differences in the CIELAB parameters among the formulations. The yogurt containing acetylated mucilage exhibited the highest L* value, indicating a lighter appearance, whereas the formulation containing native mucilage combined with gelatine showed lower luminosity. Negative a* values were observed in all samples, indicating a slight greenish hue that was more pronounced in yogurts containing native mucilage. Positive b* values indicated a tendency towards yellow tones, with higher values in formulations containing native mucilage, particularly in the presence of gelatine. Consequently, yogurts formulated with native mucilage exhibited higher chroma (C*) values, reflecting greater colour saturation, whereas those containing acetylated mucilage showed lower colour intensity. Overall, the addition of mucilage influenced the chromatic attributes of yogurts, with the effect being more noticeable in formulations containing native mucilage (*49*). The lighter appearance observed in yogurts containing acetylated mucilage may be related to structural modifications caused by the acetylation process (*51*). Additionally, the protein content of the matrix may influence chromatic stability, which could explain the variations observed in formulations containing gelatine (*52*).

Water activity (*a*_w_) ranged between 0.976 and 0.979, with no significant differences among treatments (p>0.05). The addition of mucilage and gelatine did not significantly affect this parameter. These values are consistent with those reported for commercial yogurts and, under refrigeration and acidic pH conditions, do not represent a significant microbiological risk (*53*).

Significant differences in viscosity were observed among the formulations (p<0.05). The control sample exhibited the lowest viscosity ((0.198±0.009) Pa·s). The incorporation of native mucilage increased viscosity to (0.237±0.002) Pa·s, indicating its ability to contribute to the consistency of the yogurt matrix. The combination of native mucilage and gelatine produced the highest viscosity ((0.393±0.009) Pa·s), suggesting a pronounced thickening effect associated with the combined contribution of the polysaccharide and gelatine. Hydrocolloids can modify the rheological properties of dairy systems through water binding and interactions with milk components, thereby influencing the structure and consistency of the yogurt gel (*18*). In particular, Nguyen *et al.* (*17*) reported that gelatine increased the viscosity of low-fat yogurt and reduced syneresis, which agrees with the present observation that the combination of mucilage and gelatine produced a more structured yogurt matrix.

In contrast, mucilage acetylated at 1.78 % resulted in a lower viscosity ((0.218±0.008) Pa·s) than native mucilage, which is consistent with the structural changes associated with acetylation that may alter intermolecular interactions and the hydration behaviour of polysaccharides (*51*). However, when acetylated mucilage was combined with gelatine, viscosity increased to (0.308±0.0010) Pa·s, suggesting that the combination of acetylated mucilage and gelatine contributed to the increased viscosity of the yogurt matrix (*54*). Similar effects of hydrocolloid addition on the rheological properties of low-fat yogurt have been reported by Nguyen *et al*. (*17*).

The results further showed that the incorporation of mucilage with 1.78 % acetylation at 0.5 g per 100 mL of milk improved selected technological properties of the yogurt. In particular, the formulation containing acetylated mucilage and gelatine showed higher viscosity than the control and lower syneresis, indicating improved consistency and water retention.

Regarding syneresis, the control yogurt exhibited the highest numerical value ((84.4±1.0) %). Although the yogurt containing native mucilage showed a lower value ((82.7±0.6) %), this difference was not statistically significant. In contrast, the formulations containing acetylated mucilage showed significantly lower syneresis values, reaching (80.4±0.3) % for acetylated mucilage alone and (79.9±0.4) % when combined with gelatine. The reduction in syneresis observed in these formulations is consistent with the reported ability of hydrocolloids and gelling agents to improve water retention and reduce whey separation in yogurt systems (*55*).

The effectiveness of gelatine in reducing syneresis and improving the structural properties of low-fat yogurt has also been reported by Nguyen et al. (*17*). Furthermore, previous research using *Opuntia robusta* parenchymatous tissue as a yogurt ingredient reported reduced susceptibility to syneresis, supporting the potential of *Opuntia*-derived polysaccharide-rich materials to improve water retention in yogurt systems (*12*).

A relevant aspect of the present study is that the effect of acetylation was evaluated not only at the mucilage level but also after incorporation into a low-fat yogurt matrix. Previous studies have mainly focused on the use of native cactus mucilage or other hydrocolloids, whereas the present results suggest that controlled acetylation can modify the balance between viscosity and water retention. In this study, AM 1.78 % exhibited lower viscosity than native mucilage but substantially reduced syneresis, particularly when combined with gelatine. This indicates that acetylation may provide an additional strategy for tailoring the functionality of *O. atropes* mucilage according to the technological property required in a food formulation.

Titratable acidity (as lactic acid in %) also showed significant differences among treatments (p<0.05). The formulation containing acetylated mucilage combined with gelatine exhibited the highest acidity ((0.87±0.01) %), whereas the formulation containing acetylated mucilage without gelatine showed the lowest value ((0.82±0.01) %). The control sample showed an acidity of (0.83±0.01) %, which falls within the typical range reported for natural yogurts (*53*). The higher acidity observed in formulations containing native mucilage, particularly in combination with gelatine, may be associated with increased lactose retention and enhanced bacterial activity within the gel matrix (*54*). Conversely, the lower acidity observed in the formulation containing acetylated mucilage without gelatine may be related to changes in the functional behaviour of the modified polysaccharide (*51*).

The present findings are consistent with previous reports describing the functionality of Opuntia mucilages and other plant polysaccharides as hydrocolloids in food systems (*49*). In dairy systems, the incorporation of hydrocolloids and other stabilizing components can affect water retention, gel structure, rheological properties and syneresis (*55*, *56*). The reduction in syneresis observed in this study, particularly in yogurts containing acetylated mucilage, suggests that acetylation modifies the interaction between the mucilage and the yogurt matrix. Compared with native mucilage, acetylated mucilage produced lower viscosity but a greater reduction in syneresis, indicating that thickening capacity and water-retention ability do not necessarily change in parallel. These results suggest that the functional performance of *O. atropes* mucilage depends not only on its concentration but also on its chemical structure and degree of acetylation. Therefore, controlled acetylation may provide a means of tailoring the functional properties of *O. atropes* mucilage to the technological characteristics required in a food formulation.

## CONCLUSIONS

Acetylation of *Opuntia atropes* mucilage significantly modified its physicochemical and functional properties, enhancing its potential as a natural ingredient in low-fat yogurt formulations. Optimised extraction produced a functional biopolymer from a native species with limited industrial use, while controlled acetylation, particularly at 1.78 %, improved selected technological properties, including viscosity and water absorption capacity, while maintaining oil absorption capacity. Fourier transform infrared (FTIR) spectroscopy and physicochemical analyses confirmed the structural and functional changes associated with acetylation. Incorporation of acetylated mucilage into yogurt, particularly in combination with gelatine, increased viscosity and reduced syneresis while maintaining key physicochemical parameters such as pH, titratable acidity and water activity. These results demonstrate that controlled chemical modification can be used to tailor the functionality of plant-derived polysaccharides for specific food applications.

This study also highlights the agro-industrial potential of *O. atropes* mucilage as a source of a functional plant-based hydrocolloid and demonstrates its potential application as a stabilizing ingredient in reduced-fat yogurt. Since sensory evaluation and the determination of bioactive compounds were not performed in the present study, further research should assess the sensory acceptance of the developed yogurt, particularly in terms of colour, texture and flavour, as well as the content of relevant bioactive compounds, such as phenolic compounds, and antioxidant activity before and after acetylation. These studies should be complemented by evaluations of storage stability, microbiological quality and the performance of acetylated *O. atropes* mucilage at different concentrations and under processing and storage conditions representative of industrial yogurt production. In addition, studies on process scale-up and molecular structure–function relationships would help determine the feasibility of its broader application as a food ingredient.
